# Screening Circular RNAs Related to Acquired Gefitinib Resistance in Non-small Cell Lung Cancer Cell Lines

**DOI:** 10.7150/jca.39783

**Published:** 2020-04-06

**Authors:** Chunjie Wen, Ge Xu, Shuai He, Yutang Huang, Jingjing Shi, Lanxiang Wu, Honghao Zhou

**Affiliations:** 1 Institute of Life Sciences, Chongqing Medical University, Chongqing, China; 2 Pharmacogenetics Research Institute, Institute of Clinical Pharmacology, Central South University, Changsha, China

**Keywords:** circRNA, gefitinib, acquired resistance, non-small cell lung cancer, RNA sequencing

## Abstract

**Background**: Gefitinib is a tyrosine kinase inhibitor (TKI) of epidermal growth factor receptor (EGFR) used to treat EGFR mutation-positive patients with non-small cell lung cancer (NSCLC). However, the efficacy of gefitinib is limited by the development of acquired resistance. Studies have shown that circular RNAs (circRNAs) are involved in the acquired resistance to many anticancer agents. However, the expression profiles and functions of circRNAs in gefitinib resistance in NSCLC are poorly understood so far.

**Methods**: In this study, circRNA expression profiling was explored in two gefitinib-resistant NSCLC cell lines (HCC827/GR and PC9/GR) and their parental sensitive cells (HCC827 and PC9) using high-throughput RNA sequencing. Quantitative real-time PCR (qRT-PCR) was used to confirm the expression of selected differentially expressed circRNAs. Bioinformatic tools including gene ontology (GO), Kyoto Encyclopedia of Genes and Genomes (KEGG), network analysis, and Kaplan-Meier plotter database were used to predict the functions and pathways of these differentially expressed circRNAs.

**Results**: We identified 46 and 56 differentially expressed circRNAs in HCC827/GR and PC9/GR cell lines, respectively, compared with those in their parental cell lines. Gene ontology and KEGG pathway analysis identified that the host linear transcripts of these differentially expressed circRNAs were involved in many critical biological pathways and molecular functions. We found that hsa_circ_0000567 was consistently up-regulated, and hsa_circ_0006867 was consistently down-regulated in two resistant cell lines. We further used hsa_circ_0000567 and hsa_circ_0006867 as key circRNAs to construct circRNA-miRNA-mRNA networks. Several target mRNAs of these two circRNAs had been shown to significantly associate with the overall survival of patients with lung cancer.

**Conclusions**: In this study, we generated the comprehensive expression and functional profiles of the differentially expressed circRNAs between gefitinib-resistant and -sensitive NSCLC cells, and showed that dysregulation of circRNAs might play an important role in the development of acquired resistance to gefitinib in NSCLC.

## Introduction

Lung cancer is the leading cause of cancer- related death worldwide 1. Non-small cell lung cancer (NSCLC) accounts for over 85% of primary lung cancer cases, and approximately two-thirds of patients with NSCLC are diagnosed at an advanced stage. Although the treatment of lung cancer has gradually improved, the 5-year overall survival rate is still less than 15% in patients with NSCLC 2. Epidermal growth factor receptor (EGFR) tyrosine kinase inhibitors (TKIs), such as gefitinib and erlotinib, have significantly improved the overall survival of patients with NSCLC, and are approved as first-line treatments for patients with the EGFR activating mutation 3. However, most patients will eventually develop acquired resistance after a median progression-free survival (PFS) of 8-13.7 months 3,4. Several mechanisms have been clarified, including target gene mutation, bypass signaling pathway activation and histological transformation 5. However, these mechanisms do not adequately explain the gefitinib resistance in NSCLC. Therefore, it is necessary to identify potential molecular targets and novel pathways underlying this phenomenon.

Non-coding RNA (ncRNA), such as microRNA (miRNA), long non-coding RNA (lncRNA), and circular RNA (circRNA), account for approximately 95% of the total RNAs transcribed from eukaryotic genomes, and have been shown to play an essential regulatory role in carcinogenesis 6. CircRNAs are covalently closed RNA sproduced by back-splicing events, and have numerous biological functions, such as acting as miRNA sponges, interacting with RNA-binding proteins (RBPs), as well as regulating gene transcription and splicing 7,8. CircRNAs are stable, abundant, and evolutionary conserved in mammalian cells, and often exhibit cell type-specific, tissue-specific, or developmental-stage-specific expression. Therefore, circRNAs may be ideal biomarkers for some diseases, such as cancer.

The development of high-throughput sequencing has resulted in identification of a large number of circRNAs in a wide range of solid tumors 9-14, and alterations in circRNA expression have been shown to correlate with the prognosis and chemoresistance of many cancers 15-19. For example, circKDM4C, circPAN3, and hsa_circ_0025202 have been reported to be involved in chemoresistance 20-22. These findings indicate that circRNAs may be a kind of promising biomarkers and therapeutic targets for chemoresistance. However, the roles of circRNAs in gefitinib resistance in NSCLC have not been characterized so far.

In this study, we performed high-throughput RNA sequencing in two paired gefitinib-sensitive and -resistant NSCLC cell lines, and identified differentially expressed circRNAs between the parental and resistant cells. More importantly, we showed that hsa_circ_0000567 and hsa_circ_0006867 might regulate gefitinib sensitivity by controlling the expression of many genes associated with chemoresistance. These findings improve our understanding of the mechanisms underlying acquired resistance to gefitinib, and help us to identify the potential biomarkers of gefitinib resistance in patients with NSCLC.

## Materials and Methods

### Cell culture

Human NSCLC cell lines HCC827 (EGFR E746-A750 deletion) and PC9 (EGFR exon 19 deletion) were purchased from the Cellular Institute of Chinese Academy of Science (Shanghai, China). Gefitinib- resistant cell lines (HCC827/GR and PC9/GR) were established by exposing HCC827 and PC9 cells to a gradually increasing concentration of gefitinib for 12 months as described in our previous study 23. HCC827/GR and PC9/GR cells were maintained in a medium containing 2 µM gefitinib. To eliminate the effects of gefitinib, the resistant cell lines were cultured in a drug-free medium for at least 2 weeks prior to use in experiments. All the cells were cultured in RPMI-1640 (Gibco, USA) medium supplemented with 100 U/mL of penicillin, 100 U/mL of streptomycin, and 10% fetal bovine serum (FBS) (Gibco) at 37˚C in a humidified atmosphere containing 5% CO_2_.

### Assessment of cell sensitivity to gefitinib

The cellular gefitinib sensitivity was evaluated using the MTS assay (Promega, USA). Cells were plated in 96-well plates at a density of 1×10^5^ cells/mL. After 24 h culture, the cells were treated with fresh media containing various concentrations of gefitinib (0.1-10 μmol/L) for 72 h. Then 20 μL of MTS reagent was added to each well, and the cells were incubated for 4 h at 37℃. Absorbance was measured at 490 nm using a microplate reader (BioTek, USA). Gefitinib sensitivity was evaluated using IC_50_ values (defined as the drug concentration resulting in a 50% reduction of viability compared with the control). Experiments were performed on triplicate samples from at least three independent experiments.

### RNA isolation and quality control

Total RNA was extracted from samples using TRIzol reagent (Invitrogen, USA) following the manufacturer's instruction. The quality and quantity of total RNA were measured using a NanoDrop 2000 spectrophotometer (Thermo Fisher Scientific, USA). The integrity of the RNA extracts was evaluated using denaturing a garose gel electrophoresis. Total RNA >250 ng/μL with an OD260/280 of approximately 2.0 was used (OD260/280 ranged from 1.8 to 2.0).

### RNA library construction and circRNA sequencing analysis

Total RNA from NSCLC cell lines were treated with mirVana miRNA ISOlation Kit (Ambion, USA) and RNAse R (Epicenter, USA) to remove ribosomal and linear RNA. Preparation of transcriptome libraries and sequencing were performed by Shanghai OE Biotech Co., Ltd. (Shanghai, China). Libraries were constructed using the TruSeq Stranded Total RNA Library Prep Kit (Illumina, USA) following the manufacturer's instruction and a paired-end sequencing strategy. Library quality was evaluated using a BioAnalyzer 2100 system (Agilent Technologies, USA). The libraries were sequenced using an Illumina sequencing platform (HiSeqTM 2500). For unmapped reads, the junctions were identified using a back-splicing algorithm. Differentially expressed genes and transcripts were identified by |log2(fold-change)| ≥1 and *P* ≤ 0.05 between two samples.

### Gene function analysis

The functions of circRNAs were predicated using gene ontology (GO) terms and Kyoto Encyclopedia of Genes and Genomes (KEGG) pathway enrichment analysis. GO terms analysis was performed using the DAVID gene annotation tool (http://david.abcc.ncifcrf.gov/) 24. KEGG analysis was performed to determine the involvement of target genes in different biological pathways using KOBAS software (KEGG Orthology-Based Annotation System)^25^. The threshold for GO terms and KEGG pathway enrichment analysis was *P*<0.05.

### Validation of circRNA expression

To validate the reliability of the high-throughput RNA sequencing analysis and to confirm the expression trends of circRNAs during gefitinib resistance, the expression levels of circRNAs were examined using quantitative RT-PCR (qRT-PCR). Total RNA was isolated from cell lines using TRIzol reagent and reverse transcribed into cDNA using the AMV reverse transcription kit (Promega, USA). qRT-PCR was performed using SYBR Premix Ex Taq (Takara, Japan), and GAPDH was used as an internal control. The expression of circRNAs was defined based on the threshold cycle (Ct), and relative expression levels were calculated using the 2^-ΔΔCt^ method. The PCR thermal programs were as follows: 95℃ for 30 sec followed by 40 cycles of 95℃ for 5 sec, then 60℃for 30 sec. Primer sequences for candidate genes are listed in Table S1.

### Construction of the circRNA-miRNA-mRNA network

CircRNA-miRNA interactions were predicted using Arraystar's home-made miRNA target prediction software based on miRanda (http://www.microrna.org/) 26. Cytoscape software (The Cytoscape Consortium, NY) was used to generate a circRNA-miRNA-mRNA interaction network based on the correlation analysis 27.

### Survival analysis

The prognostic significance of the mRNA expression profiles in NSCLC was evaluated using the Kaplan-Meier plotter (www.kmplot.com), an online database that includes gene expression data and survival information downloaded from Gene Expression Omnibus (GEO). We analyzed the overall survival (OS) data of 1,928 lung cancer patients downloaded from GEO using a Kaplan-Meier survival plot. The mRNAs were uploaded into the database to obtain Kaplan-Meier survival plots, in which the number at risk was shown below the main plot. Log rank *P*-values and hazard ratios (HR) with 95% confidence intervals were calculated and displayed by the software. Log-rank *P*-values < 0.05 were considered statistically significant 28.

### Statistical analysis

Experimental data are represented as the mean ± SEM of a minimum of three biological replicates. Student's two-tailed unpaired *t*-test was used to determine differences between two groups in *in vitro* experiments. *P*-values < 0.05 were considered statistically significant. All statistical analyses were performed using GraphPad Prism 5.0 software (GraphPad, Inc., USA).

## Results

### Validation of gefitinib resistance in the HCC827/GR and PC9/GR cell lines

**Figure 1:**
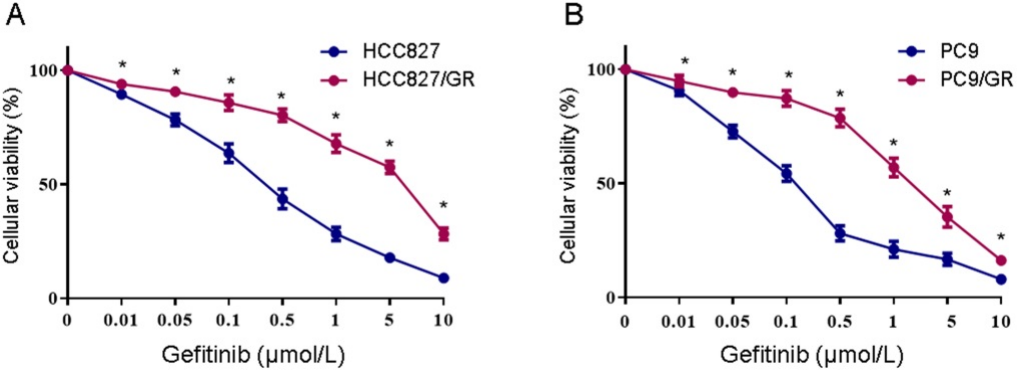
** Determination of gefitinib sensitivity of NSCLC cell lines. (A)** MTS assays were conducted in the gefitinib-resistant HCC827/GR and gefitinib-sensitive HCC827 cell lines. **(B)** MTS assays were conducted in the gefitinib-resistant PC9/GR and gefitinib-sensitive PC9 cell lines. Data are presented as the mean ± SEM (n = 3). *P < 0.05 compared with the parental cell lines.

Two NSCLC cell lines with acquired resistance to gefitinib, HCC827/GR and PC9/GR, were derived from their parental cells through continuous exposure to increasing concentrations of gefitinib over 12 months. The gefitinib sensitivity was verified using the MTS assay. As shown in Figure 1, after 48 h of gefitinib exposure, the IC_50_ values were 0.29 ± 0.049 μM and 0.11 ± 0.04 μM in HCC827 and PC9 cells, respectively. As expected, HCC827/GR and PC9/GR cells showed significantly decreased sensitivity to gefitinib, with the IC_50_ values of 2.89 ± 0.2 μM and 1.8 ± 0.08 μM, respectively. These results indicate that the HCC827/GR and PC9/GR cells are more resistant to gefitinib than their parental cell lines.

### Overview of circRNAs profiles

**Figure 2:**
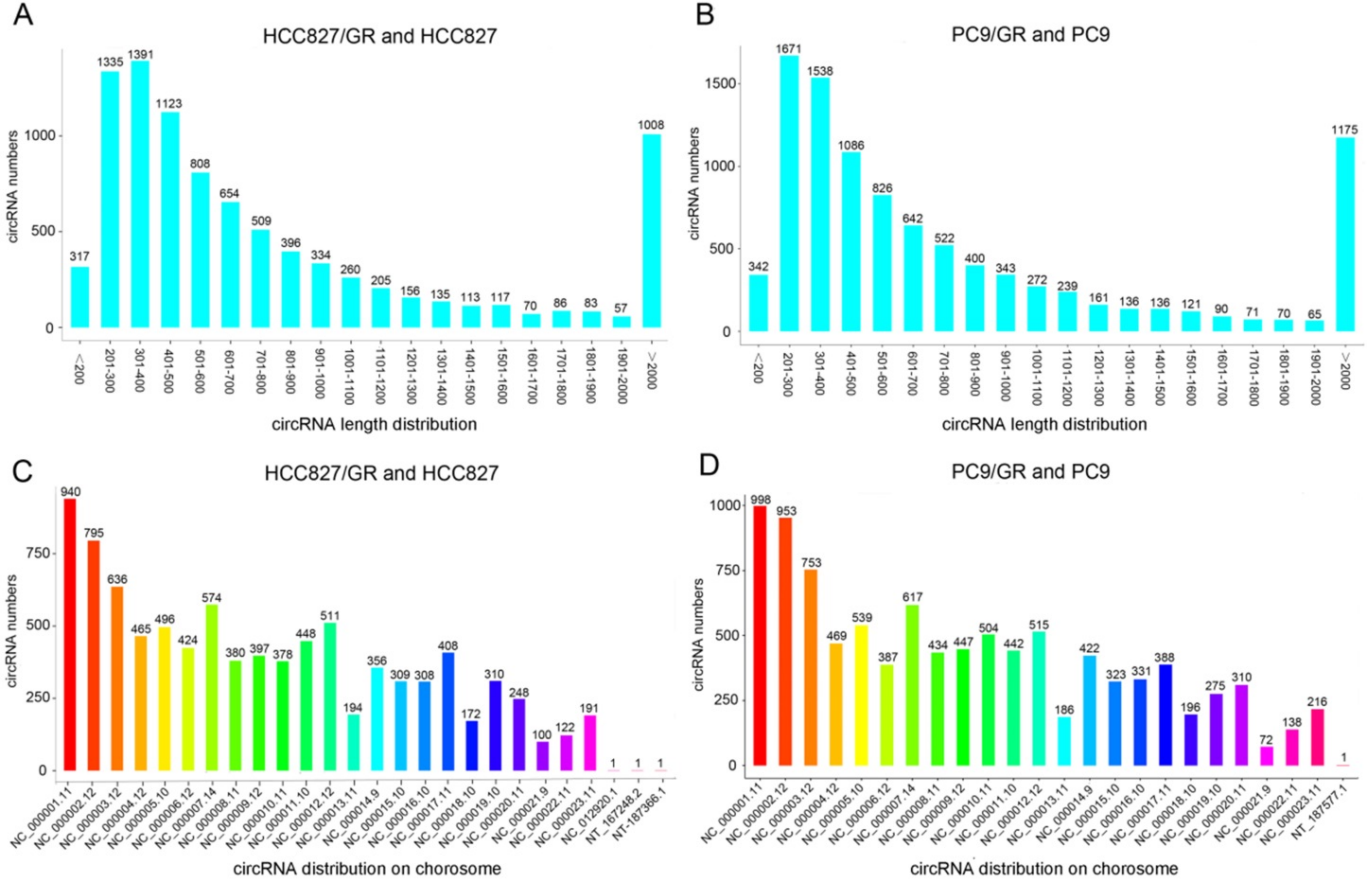
** Overview of circRNA profiles. (A and B)** The length distribution of circRNAs. **(C and D)** Chromosome distribution of circRNAs.

**Figure 3:**
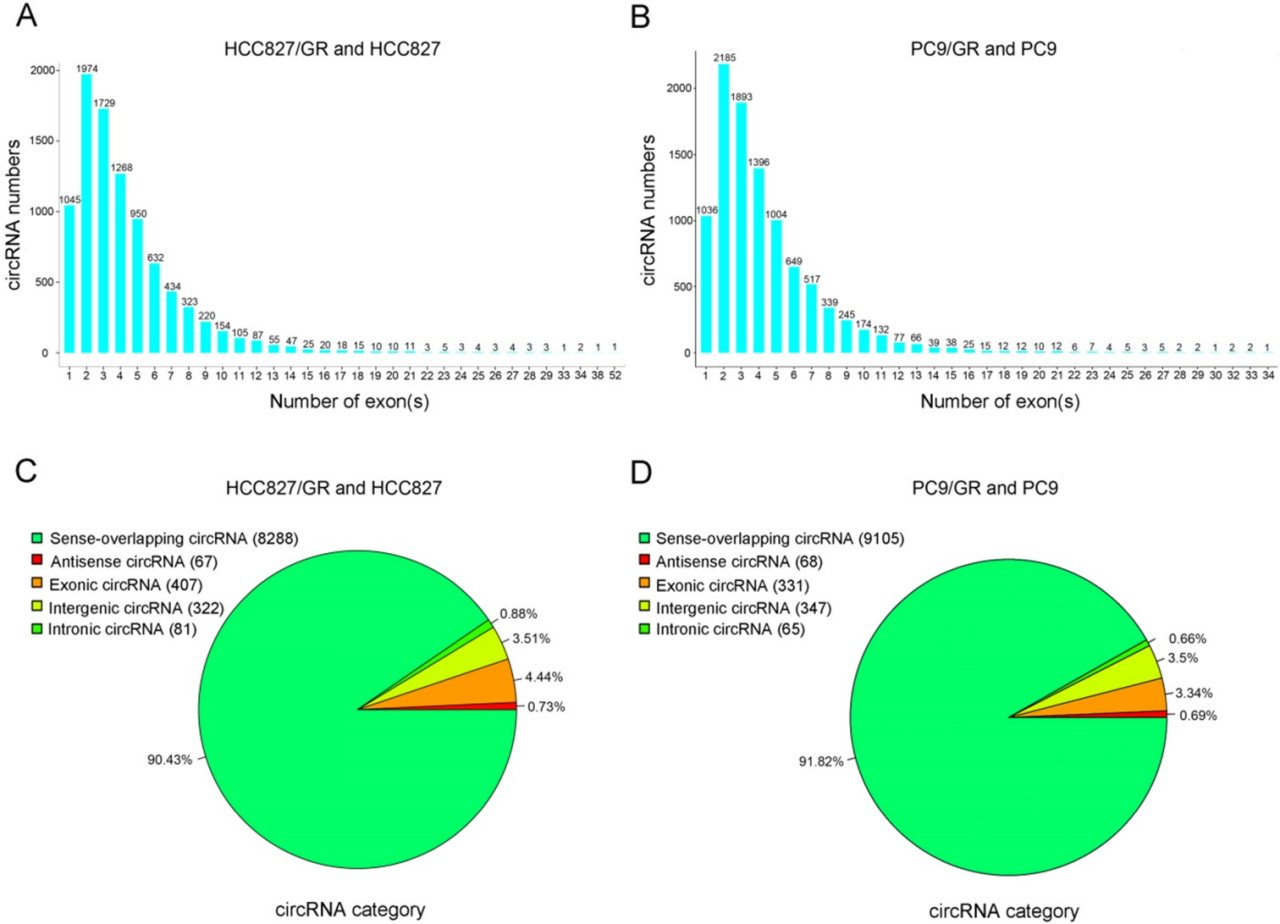
** Overview of circRNA profiles. (A and B)** Distribution of the exon numbers of circRNAs. **(C and D)** Category of circRNAs based on genomic origin.

We performed ribosomal RNA-depleted RNA sequencing to explore circRNA expression profiles in two paired gefitinib-sensitive and -resistant NSCLC cell lines. A total of 9,157 circRNAs were detected in HCC827/GR and HCC827 group, and 9,906 circRNAs were identified in PC9/GR and PC9 group. The sizes of the identified circRNA candidates ranged from under 200 nucleotides (nt) to over 2,000 nt, with the majority between 200 and 1,000 nt (Figure 2A and 2B). These circRNAs originated from all chromosomes, chr1, chr2, and chr3 were the three chromosomes to which the most circRNAs were mapped (Figure 2C and 2D). The number of exons per circRNA was less than six for most circRNAs (Figure 3A and 3B). The identified circRNAs included antisense, exonic, intronic, intergenic, as well as sense-overlapping circRNAs (Figure 3C and 3D). Among them, sense- overlapping circRNAs were most abundant, which suggests that these circRNAs may be particularly important.

### Identification of differentially expressed profiles of circRNAs between gefitinib -sensitive and -resistant NSCLC cell lines

**Figure 4:**
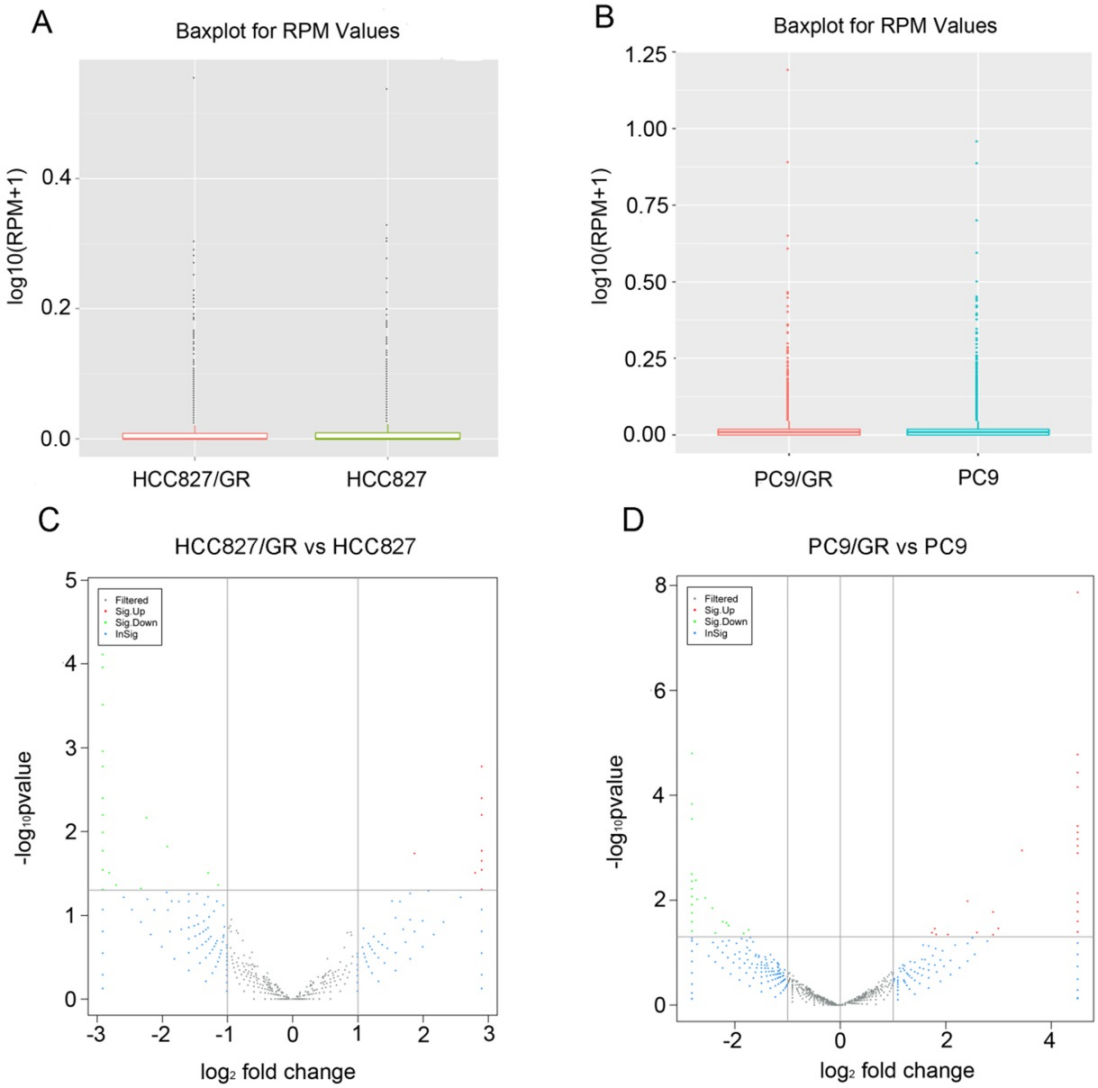
** Expression patterns of circRNAs. (A and B)** Box plots showed that the distributions of circRNAs intensities from all normalized datasets. **(C and D)** Volcano plots indicated the variation of circRNA expression with statistical significance between gefitinib-resistance and -sensitive NSCLC cells.

The distribution of circRNA intensities from all the normalized datasets is shown in box plots, and the results demonstrated that no abnormal expression was observed in all the cells (Figure 4A and Figure 4B). Volcano plots showed that the profile of circRNA expression was significantly different between the resistant and sensitive cells (Figure 4C and Figure 4D). Using cutoff fold changes ≥2 or ≤0.5, and a false discovery rate < 0.5, a total of 46 circRNAs were found to be differentially expressed between HCC827/GR and HCC827 cells. Among them, 11 were up-regulated and 35 were down-regulated in HCC827/GR cells (Table S2). Fifty-six circRNAs were differentially expressed between PC9/GR and PC9 cells, among which 26 were up-regulated and 30 were down-regulated in PC9/GR cells (Table S3). Furthermore, we found that hsa_circ_0000567 was consistently up-regulated and hsa_circ_0006867 was consistently down-regulated in both resistant cell lines compared to their parental cells (Table 1). These two circRNAs may play an important role in the development of gefitinib resistance.

### Prediction of biological functions of differentially expressed circRNAs

**Figure 5:**
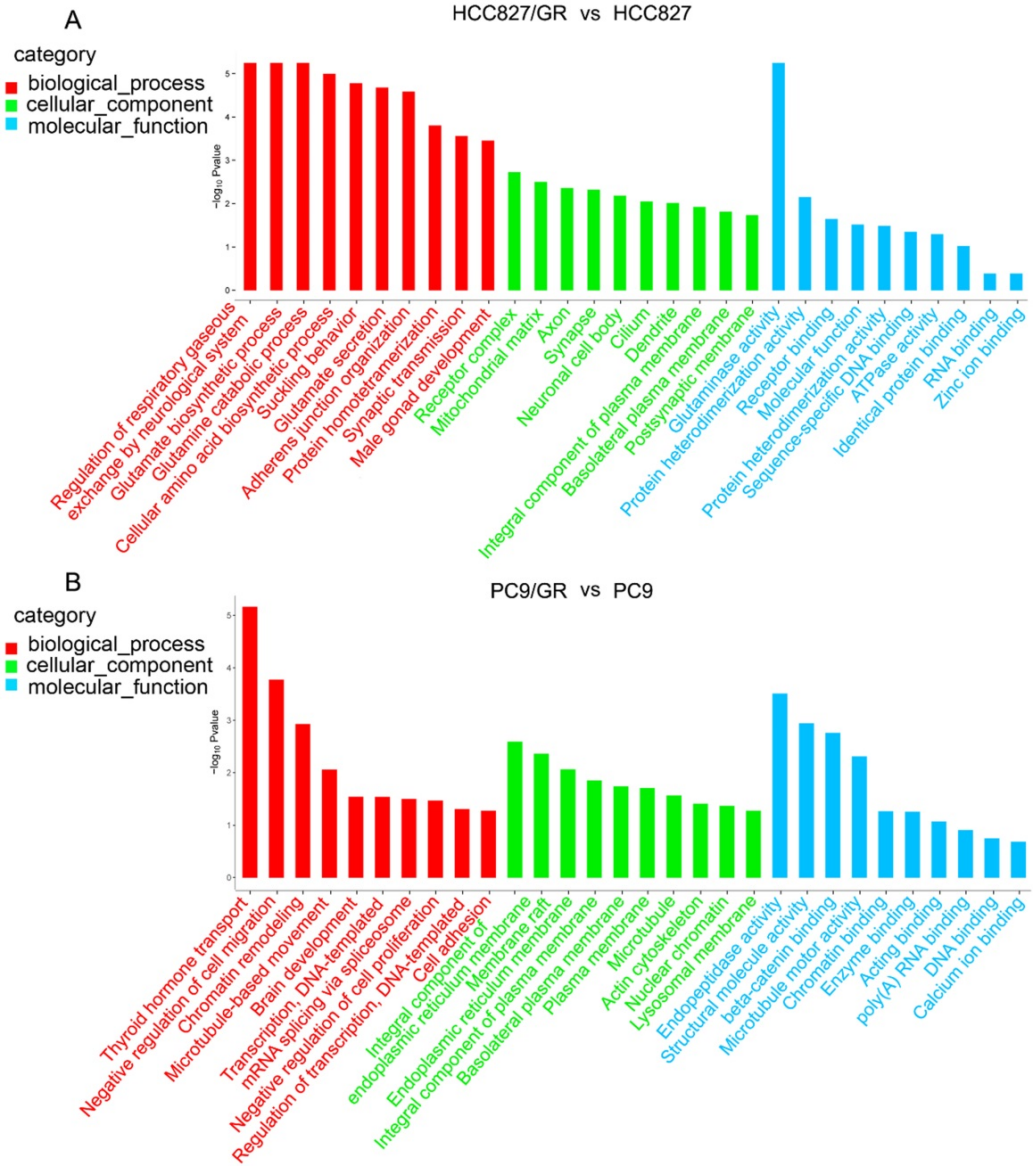
** Gene ontology annotations of the host linear transcripts of differentially expressed circRNAs. (A)** Gene ontology analyses of the host linear transcripts of differentially expressed circRNAs in HCC827/GR *vs* HCC827 group. **(B)** Gene ontology analyses of the host linear transcripts of differentially expressed circRNAs in PC9/GR *vs* PC9 group.

To further explore the functions of these differentially expressed circRNAs, we performed GO analysis and KEGG pathway analysis on their host linear transcripts. The results showed that compared with their parental cells, there were 227 and 411 GO terms were up-regulated in HCC827/GR and PC9/GR cells, respectively. A total of 555 and 291 GO terms were down-regulated in HCC827/GR and PC9/GR cells, respectively. GO analyses covered three subgroups: biological process (BP), cellular component (CC), and Molecular function (MF). In HCC827/GR *vs* HCC827 group, the most significantly enriched GO terms in the BP, CC, and MF subgroups were regulation of respiratory gaseous, receptor complex, and glutaminase activity, respectively. In PC9/GR *vs* PC9 group, the most significantly enriched GO terms in the BP, CC, and MF subgroups were thyroid hormone transport, integral component of endoplasmic reticulum membrane, and endopeptidase activity, respectively (Figure 5). KEGG pathway analysis showed that central carbon metabolism in cancer was the most significantly associated pathway in HCC827/GR* vs* HCC827 group (Table 2). While the most enriched pathway in PC9/GR *vs* PC9 group was bile secretion (Table 3).

### Validation of differentially expressed circRNAs between gefitinib-resistant and -sensitive NSCLC cell lines

**Figure 6:**
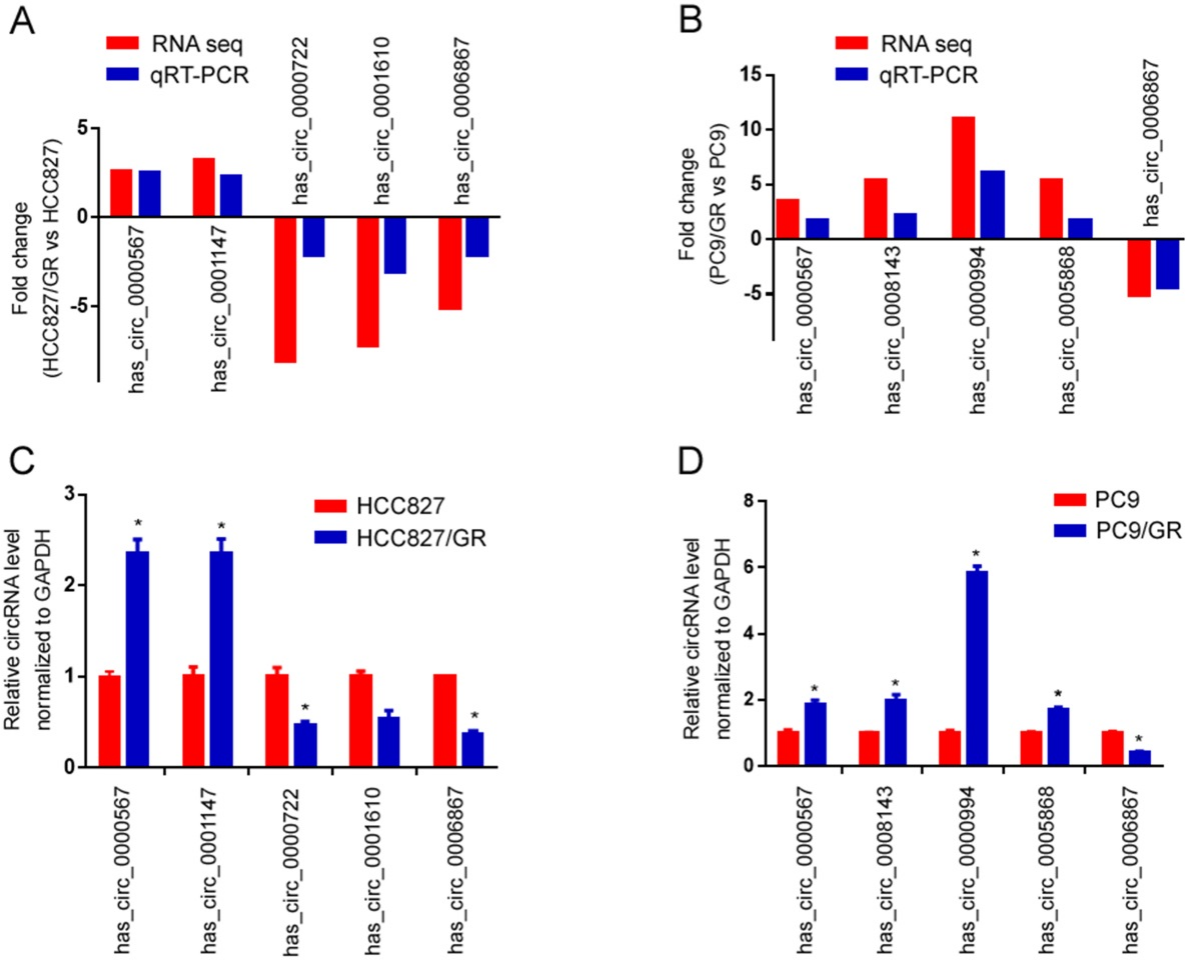
** Validation of RNA sequencing data using qRT-PCR. (A)** Compared with the expression of five significantly different expressed circRNAs using sequencing and qRT-PCR analysis between HCC827/GR and HCC827 cell lines. **(B)** Compared with the expression of five significantly different expressed circRNAs using sequencing and qRT-PCR analysis between PC9/GR and PC9 cell lines. **(C)** Validating the expression of the five significantly different expressed circRNAs in HCC827/GR cell lines compared with HCC827 cell lines. **(D)** Validating the expression of the five significantly different expressed circRNAs in PC9/GR cell lines compared with PC9 cell lines. Data are presented as the mean ± SEM (n = 3). *P < 0.05 compared with the parental cell lines.

To validate the high-throughput RNA sequencing results, we selected five differentially expressed circRNAs in each group (HCC827/GR *vs* HCC827, PC9/GR *vs* PC9 group) for further validation by qRT-PCR. Among these circRNAs, hsa_circ_0000567 and hsa_circ_0006867 were consistently dysregulated in both groups, while hsa_circ_0001147, hsa_circ_0000722, hsa_circ_0001610, hsa_circ_0008143, hsa_circ_0000994 as well as hsa_circ_0005868 were randomly chosen. As shown in Figure 6A and Figure 6B, the qRT-PCR results were highly consistent with those of the sequencing. Furthermore, hsa_circ_0000567 was consistently up-regulated in both resistant cell lines compared to their parental cells, whereas hsa_circ_0006867 was consistently down-regulated in both resistant cells (Figure 6C and 6D).

### CircRNA-miRNA-mRNA networks

**Figure 7:**
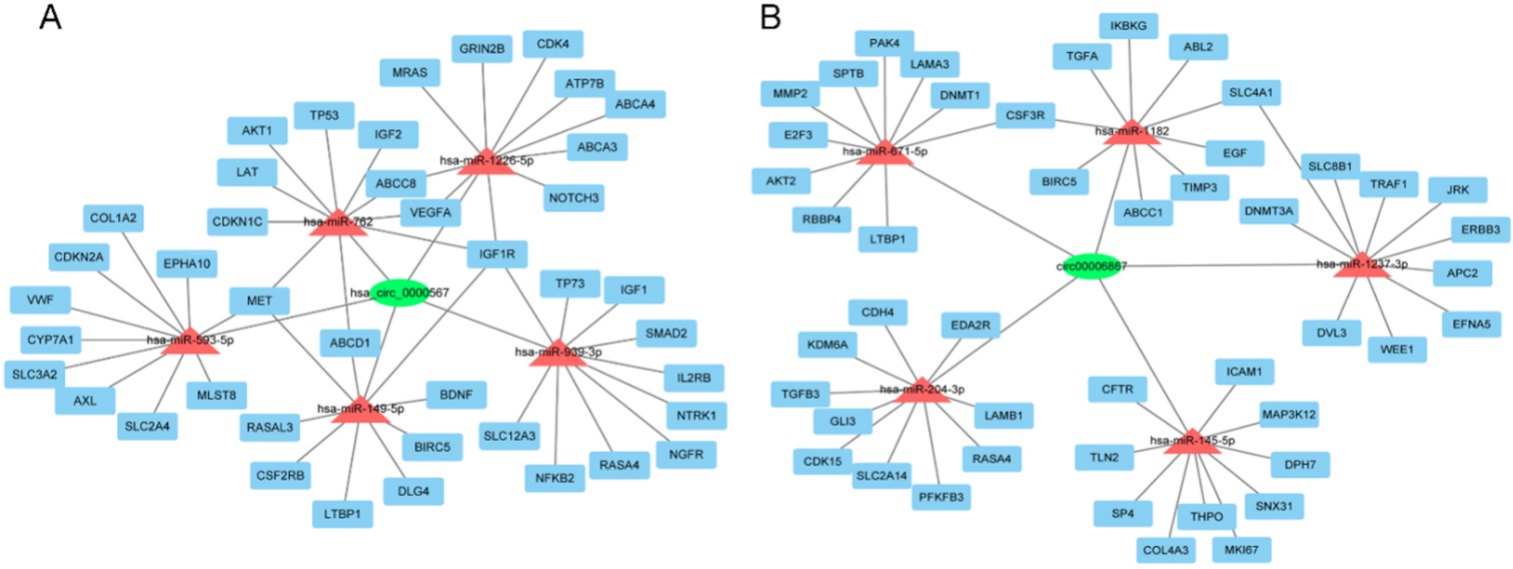
** The circRNA-miRNA-mRNA network. (A)** Interaction network of hsa-circ-0000567. **(B)** Interaction network of hsa-circ-0006867.

To further evaluate the roles of hsa_circ_0000567 and hsa_circ_0006867 in gefitinib resistance in NSCLC, we assumed that these circRNAs acted as miRNA sponges and played a role in circRNA- miRNA-mRNA interaction networks. We identified the top 5 miRNAs that could potentially bind to these circRNAs, as well as their 10 target mRNAs (Figure 7A and Figure 7B). Many of these target mRNAs, such as MET, AKT1, ABCB1, and IGF1R have been identified as key players in resistance to gefitinib^29^-^32^. The network map showed the potential target mRNAs of hsa_circ_0000567 and hsa_circ_0006867, and provided information regarding potential mechanisms of these circRNAs in acquired resistance to gefitinib in NSCLC.

Then, we analyzed the correlation between the target mRNAs in circRNA-miRNA-mRNA networks with the overall survival (OS) of lung cancer patients. The OS of 1,928 patients without any classification were analyzed using the online database www.kmplot.com/lung cancer. We performed univariate Cox regression analysis based on the expression value of these mRNAs. We found that 21 target mRNAs of hsa_circ_0000567 and 19 target mRNAs of hsa_circ_0006867 were closely related to the OS of patients. High levels of ABCC8, ABCD1, BDNF, BIRC5, CDK4, CDKN2A, DLG4, GRIN2B, IGF1R, MET, MLST8, NFKB2, NGFR, NOTCH3, RASA4, SLC2A4, SLC3A2, SLC12A3, TP53, TP73, VEGFA, as well as low levels of ABL2, AKT2, CDK15, CFTR, DNMT3A, EFNA5, ERBB3, GLI3, ICAM1, JRK, KDM6A, LAMA3, LAMB1, LTBP1, MAP3K12, PFKFB3, RBBP4, SP4, TIMP3 were associated with poor survival in patients with lung cancer (Figure S1 and S2). These results indicate that hsa_circ_0000567 and hsa_circ_0006867 as well as their downstream mRNAs are potentially associated with the overall survival of patients with lung cancer.

## Discussion

EGFR-TKIs, such as gefitinib (ZD1839, Iressa) and erlotinib (OSI-774, Tarceva), are the standard treatment for NSCLC with advanced EGFR mutation. However, almost all cases experience disease recurrence after 1 to 2 years due to acquired resistance 3, 4. However, the mechanisms still not fully elucidated.

Previous studies have established that circRNAs play an important role in cancer onset and development. Aberrant circRNA expression patterns have been described in various type of cancers 9-14, and alterations in circRNA expression have been shown to correlate with cancer prognosis and chemotherapy resistance 15-19, 33. For example, overexpressed circPVT1 contributes to doxorubicin and cisplatin resistance of osteosarcoma cells by regulating ABCB1 34. CircPAN3 mediates drug resistance in acute myeloid leukemia through the miR-153-5p/miR-183-5p-XIAP axis 16. CircRNA101505 sensitizes hepatocellular carcinoma cells to cisplatin by sponging miR-103 and enhancing oxidored-nitro domain-containing protein 1 expression 35. However, the expression profiles and functions of circRNA in gefitinib resistance in NSCLC are poorly understood.

In the present study, we characterized the circRNA expression patterns of two paired gefitinib-resistant and -sensitive NSCLC cell lines using high-throughput RNA sequencing. The length of most circRNA was around 200-1000 nt, which consistent with previous reports showing the median length of circRNAs is around 500 nt 36. CircRNAs are primarily generated from exons or introns of their host linear transcripts, and are involved in regulation of their host genes expression 37,38. Therefore, after we screened out the differentially expressed circRNAs between gefitinib-resistant and sensitive cells, we predicted the biological functions of their host linear transcripts using GO and KEGG pathway analyses. Several pathways identified in our analysis have been reported to be involved in chemoresistance. In the HCC827/GR cell line, the most significantly enriched pathway was central carbon metabolism in cancer. Stauber *et al.* found that drug resistance in leukemia cells was associated with this pathway 39. In PC9/GR cells, bile secretion was the most significantly enriched pathway. A previous study showed that bile acids could increase doxorubicin sensitivity through regulation of ABCC1 40. However, the functions of host linear transcripts could not fully reflect the functions of circRNAs. Recent studies have demonstrated that circRNAs can function as a microRNA (miRNA) or RNA binding protein sponge, and regulate splicing or transcription 41.

Two key circRNAs (hsa_circ_0000567 and hsa_circ_0006867) identified during screening and verified using qRT-PCR were selected for further analysis. CircRNA-miRNA-mRNA axis involving hsa_circ_0000567 and hsa_circ_0006867 were predicted. Analysis using Cytoscape predicted that hsa_circ_0000567 could sponge to hsa-miR-1226-5p, hsa-miR-762, hsa-miR-593-5p, hsa-miR-149-5p, and hsa-miR-939-3p. Previous reports have demonstrated that these predicted miRNAs were associated with either cancer development or chemoresistance. For example, miR-1226, miR-593-5p, and miR-939 have been shown to play a role in acquired resistance to tamoxifen, cisplatin, and 5-fluorouracil, respectively 42-44. While miR-762 and miR149-5p are involved in promoting the progression of breast cancer and NSCLC, respectively 45,46. In addition, the top 5 candidate miRNAs which could be sponged by hsa_circ_0006867 were hsa-miR-204-3p, hsa-miR-671- 5p, hsa-miR-145-5p, hsa-miR-1237-3p, and hsa-miR- 1182. Among them, the level of miR-145 has been shown to correlate highly with the efficacy of 5-fluorouracil in colorectal cancer 47.

We also predicted the roles of circRNAs through evaluating the functions of target mRNAs in our constructed circRNA-miRNA-mRNA networks. Several mRNAs have been reported to be involved in gefitinib resistance in NSCLC. For example, the amplification of MET gene, which potentially was modulated by hsa-miR-593-5p, hsa-miR-762, or hsa-miR-149-5p in our miRanda analysis, accounts for 5% to 10% of cases of EGFR-TKI resistance 29. In addition, AKT1, ABCB1, and IGF1R have also been shown to also associate with gefitinib resistance in NSCLC 30-32. Although a cohort analysis of the association between these mRNAs and gefitinib resistance is not currently available, but our analysis of OS showed that both the up-regulation of ABCC8, ABCD1, BDNF, BIRC5, CDK4, CDKN2A, DLG4, GRIN2B, IGF1R, MET, MLST8, NFKB2, NGFR, NOTCH3, RASA4, SLC2A4, SLC3A2, SLC12A3, TP53, TP73, VEGFA, and the down-regulation of ABL2, AKT2, CDK15, CFTR, DNMT3A, EFNA5, ERBB3, GLI3, ICAM1, JRK, KDM6A, LAMA3, LAMB1, LTBP1, MAP3K12, PFKFB3, RBBP4, SP4, TIMP3 were associated with poor prognosis of patient with lung cancer. Of these mRNAs, NOTCH3 and IGF1R are associated with EGFR-TKI resistance 48,32. Other target mRNAs, such as SLC3A2, CDK4 and CDKN2A are also closely related to chemoresistance 49-51. These data indicates that we successfully construct a cancer-associated circRNA-miRNA-mRNA network for lung cancer. Meanwhile, our further research will focus on the relationship between selected circRNAs and their target miRNAs and mRNAs with *in vivo* and *in vitro* experiments.

In conclusion, our study characterized the expression profiles of circRNAs after gefitinib resistance in NSCLC cells, and showed that circRNAs might play a role in gefitinib resistance in NSCLC. Furthermore, hsa_circ_0000567 and hsa_circ_00006867 might be candidates for further verification and functional analysis.

## Supplementary Material

Supplementary figures and tables.

## Figures and Tables

**Table 1:** Two dysregulated circRNAs potentially related to acquired resistance to gefitinib

circRNA ID	Gene ymbol	Regulation	circRNA_chr	circRNA_ length/bp
hsa_circ_0000567	SETD3	Up	NC_000014.9	683
hsa_circ_0006867	LRBA	Down	NC_000004.12	263

**Table 2:** KEGG analysis of host linear transcripts in HCC827/GR cells compared to HCC827 cells

Pathway	*P*-value	Pathway name
Path:hsa05230	3.08E-06	Central carbon metabolism in cancer
path:hsa00220	3.24E-06	Arginine biosynthesis
path:hsa00471	3.24E-06	Proximal tubule bicarbonate reclamation
path:hsa04724	5.68E-05	Glutamatergic synapse
path:hsa00250	8.12E-05	Alanine, aspartate and glutamate metabolism

**Table 3:** KEGG analysis of host linear transcripts in PC9/GR cells compared to PC9 cells

Pathway	*P*-value	Pathway name
path:hsa04976	0.000397	Bile secretion
path:hsa04670	0.008397	Leukocyte transendothelial migration
path:hsa04510	0.009555	Focal adhesion
path:hsa04141	0.009873	Protein processing in endoplasmic reticulum
path:hsa03013	0.012765	RNA transport
