# Supplementary material for: Screening Circular RNAs Related to Acquired Gefitinib Resistance in Non-small Cell Lung Cancer Cell Lines
Source: J Cancer. 2020 Apr 6;11(13):3816–26. doi: 10.7150/jca.39783 (PMC7171488; doi:10.7150/jca.39783)

Table S1. The list of primers (F, forward and R, reverse) used for qRT-PCR validation

Gene	Primer sequence
**has_circ_0000567**	F: 5'-TTCGGTATCTTCAGTCCACACA-3'<br>R: 5'-TTCCTTTGGTGACACAGTTGC-3'
**has_circ_0000722**	F: 5'-GCGCCATCCTCCAGCTTT-3'<br>R: 5'-GCGGTGGAAAGCATCCCTA-3'
**has_circ_0001147**	F: 5'-TGCTGGACCTATGAGGCTTT-3'<br>R: 5'-TGTCCTTGCATCTCTTTCCTCA-3'
**has_circ_0005868**	F: 5'-GAGGAATACATCAGAGACAAT-3'<br>R: 5'-GTTGAAAGGAATCCTCAAAC-3'
**has_circ_0006867**	F: 5'-CACGATAGCAGGACGGAGTTA-3'<br>R: 5'-TGCAGCCCTTCTGTATATGCC-3'
**has_circ_0000994**	F: 5'-TCGAGGAAATGTTATCGTTCCA-3'<br>R: 5'-TCCAACTGTCACAACCTAACAAT-3'
**has_circ_0008143**	F: 5'-AGCTCTACAGATGAGTGGCT-3'<br>F: 5'-CCAGCTGCATACAGAAGACTG-3'
**has_circ_0001610**	F: 5'- CCAGAACCGGGAGAAATGGA-3'<br>R: 5'- TGGCGGTATGTGCCAATGA-3'
**GAPDH**	F: 5'-GGATGCAGGAGATCACTG-3'<br>R: 5'-CGATCCACACGGAGTACTT-3'

Table S2. The significantly up-regulated and down-regulated circRNAs in the HCC827/GR cells compared with HCC827 cells

ID	baseMean (HCC827)	baseMean (HCC827/GR)	Foldchange	P value	Up-Down	type	Gene
hsa_circ_0005695	0	9.547365	Inf	0.028634	Up	sense-overlapping	SLC30A6
hsa_circ_0055151	0	13.348117	Inf	0.004004	Up	sense-overlapping	ZNF638
hsa_circ_0001093	0	8.275686	Inf	0.04912	Up	exonic	ALS2
hsa_circ_0003337	0	8.13082	Inf	0.04912	Up	antisense	NOP58
hsa_circ_0066776	0	10.13082	Inf	0.004004	Up	sense-overlapping	PVRL3
hsa_circ_0001410	0	13.05839	Inf	0.003058	Up	sense-overlapping	DCUN1D4
hsa_circ_0001633	0	10.275686	Inf	0.016977	Up	sense-overlapping	SOBP
hsa_circ_0007434	0	8.348117	Inf	0.04912	Up	sense-overlapping	SMURF2
hsa_circ_0081964	2.036818	14.348117	7.044378	0.031094	Up	sense-overlapping	IMMP2L
hsa_circ_0001147	3.16741	10.275686	3.244192	0.015755	Up	sense-overlapping	RBM39
hsa_circ_0000567	42.752936	98.54639	2.305017	0.02801	Up	sense-overlapping	SETD3
hsa_circ_0000231	114.765467	52.348117	0.456131	0.04352	Down	sense-overlapping	ARHGAP12
hsa_circ_0001900	83.86329	34.841165	0.415451	0.031148	Down	sense-overlapping	CAMSAP1
hsa_circ_0032649	34.075361	9.275686	0.27221	0.015124	Down	exonic	MLH3
hsa_circ_0001725	15.156175	3.98596	0.262992	0.047786	Down	sense-overlapping	LMTK2
hsa_circ_0023919	15.156175	3.05839	0.201791	0.047786	Down	sense-overlapping	PICALM
hsa_circ_0006867	13.234262	2.6238	0.198258	0.043469	Down	sense-overlapping	LRBA
hsa_circ_0002439	14.156175	2.275686	0.160755	0.031094	Down	sense-overlapping	TFAP4
hsa_circ_0001610	45.156175	6.275686	0.138977	0.000306	Down	sense-overlapping	TNFRSF21
hsa_circ_0000722	16.624699	2.069112	0.12446	0.001101	Down	exonic	GSE1
hsa_circ_0000179	11.512459	0	0	0.010264	down	sense-overlapping	DTL
hsa_circ_0057123	8.65722	0	0	0.04912	Down	sense-overlapping	CDCA7
hsa_circ_0004191	8.31235	0	0	0.04912	Down	sense-overlapping	PGAP1
hsa_circ_0001277	8.31235	0	0	0.04912	Down	sense-overlapping	SLC4A7
hsa_circ_0005605	8.390437	0	0	0.04912	Down	sense-overlapping	TBC1D23
hsa_circ_0067492	12.390437	0	0	0.006339	Down	sense-overlapping	IL20RB
hsa_circ_0002785	12.85896	0	0	0.006339	Down	sense-overlapping	ADGRA3
hsa_circ_0006916	9.40557	0	0	0.028634	Down	sense-overlapping	HOMER1
hsa_circ_0004973	8.78087	0	0	0.04912	Down	sense-overlapping	MTO1
hsa_circ_0001638	8.546612	0	0	0.04912	Down	sense-overlapping	KIAA1919
hsa_circ_0007618	15.18645	0	0	0.001674	Down	sense-overlapping	DOCK5
hsa_circ_0085173	8.624699	0	0	0.04912	Down	sense-overlapping	DENND4C
hsa_circ_0008342	9.17131	0	0	0.028634	Down	sense-overlapping	CLNS1A
hsa_circ_0023812	10.546612	0	0	0.016977	Down	sense-overlapping	NARS2
hsa_circ_0006128	8.546612	0	0	0.04912	Down	sense-overlapping	AEBP2
hsa_circ_0004047	8.546612	0	0	0.04912	Down	sense-overlapping	BAZ1A
hsa_circ_0000557	8.624699	0	0	0.04912	Down	sense-overlapping	NEK9
hsa_circ_0000642	8.78087	0	0	0.04912	Down	sense-overlapping	ZFAND6
hsa_circ_0006127	9.546612	0	0	0.028634	Down	sense-overlapping	SRCAP

hsa_circ_0007766	13.702787	0	0	0.004004	Down	sense-overlapping	ERBB2
hsa_circ_0004956	8.702787	0	0	0.04912	Down	sense-overlapping	UBA2
hsa_circ_0059475	8	0	0	0.04912	Down	sense-overlapping	TASP1
hsa_circ_0002805	8	0	0	0.04912	Down	sense-overlapping	ZBTB46
hsa_circ_0001189	9	0	0	0.028634	Down	sense-overlapping	MORC3
hsa_circ_0003102	10	0	0	0.016977	Down	sense-overlapping	LRP5L
hsa_circ_0001246	9	0	0	0.028634	Down	sense-overlapping	ATXN10

Table S3. The significantly up-regulated and down-regulated circRNAs in the PC9/GR cells compared with the PC9 cells

ID	BaseMean (PC9)	BaseMean (PC9/GR)	Fold change	p-value	Regulation	Type	Gene
hsa_circ_0013743	0	11.386081	Inf	0.016477	Up	sense-overlapping	MAN1A2
hsa_circ_0000145	0	9.315885	Inf	0.040286	Up	sense-overlapping	PFDN2
hsa_circ_0058495	0	20.70197	Inf	0.000684	Up	sense-overlapping	RHBDD1
hsa_circ_0087220	0	13.45628	Inf	0.007352	Up	sense-overlapping	C9orf41
hsa_circ_0000471	0	22.77216	Inf	0.000387	Up	sense-overlapping	N4BP2L2
hsa_circ_0030388	0	12.42118	Inf	0.010919	Up	intergenic	
hsa_circ_0000530	0	9.315885	Inf	0.040286	Up	sense-overlapping	CTAGE5
hsa_circ_0006886	0	18.63177	Inf	0.001246	Up	sense-overlapping	FAM192A
hsa_circ_0007580	0	9.315885	Inf	0.040286	Up	sense-overlapping	PRKCA
hsa_circ_0000799	0	9.315885	Inf	0.040286	Up	sense-overlapping	BPTF
hsa_circ_0061651	0	9.315885	Inf	0.040286	Up	exonic	HLCS
hsa_circ_0007817	0	10.35098	Inf	0.025361	Up	sense-overlapping	HUWE1
hsa_circ_0002153	0	9.315885	Inf	0.040286	Up	sense-overlapping	MID2
hsa_circ_0008901	3.864367	87.983358	22.76786	1.67-05	Up	sense-overlapping	PTPN12
hsa_circ_0000994	3.864367	42.43903	10.98214	0.001123	Up	exonic	SLC8A1
hsa_circ_0007528	1.932184	15.52648	8.035714	0.034554	Up	sense-overlapping	PIGN
hsa_circ_0003511	2.898275	21.73707	7.5	0.016725	Up	sense-overlapping	LMBR1
hsa_circ_0000914	1.932184	14.49138	7.5	0.045578	Up	sense-overlapping	FKBP8
hsa_circ_0006837	2.898275	17.59667	6.071429	0.041192	Up	sense-overlapping	RERE
hsa_circ_0002458	8.694826	46.57943	5.357143	0.010377	Up	sense-overlapping	PTPN12
hsa_circ_0005868	2.898275	15.52647	5.357143	0.036569	Up	sense-overlapping	ITCH
hsa_circ_0008143	1.932184	10.35098	5.357139	0.025361	Up	sense-overlapping	RBM39
hsa_circ_0002191	6.762642	27.94766	4.132653	0.045229	Up	sense-overlapping	C9orf3
hsa_circ_0000567	12.55919	44.50923	3.543956	0.044997	Up	sense-overlapping	SETD3
hsa_circ_0083709	23.1862	80.73767	3.482143	0.034864	Up	sense-overlapping	DOCK5
hsa_circ_0088030	22.22011	74.52708	3.354037267	0.041622	Up	sense-overlapping	PTGR1
hsa_circ_0006867	9.660918	4.140393	0.428571	0.038795	Down	sense-overlapping	LRBA
hsa_circ_0031584	154.5747	46.57943	0.301339	0.036748	Down	sense-overlapping	ARHGAP5
hsa_circ_0009061	51.20286	14.49138	0.283019	0.04295	Down	sense-overlapping	KDM1A
hsa_circ_0006410	35.7454	8.280787	0.23166	0.030249	Down	sense-overlapping	TUSC3
hsa_circ_0016601	36.71149	8.280787	0.225564	0.026985	Down	sense-overlapping	DNAH14
hsa_circ_0008282	33.81321	7.245688	0.214286	0.02571	Down	sense-overlapping	MARK3
hsa_circ_0003848	21.25402	4.140393	0.194805	0.042271	Down	sense-overlapping	PSEN1
hsa_circ_0032649	38.64367	7.245688	0.1875	0.014126	Down	exonic	MLH3
hsa_circ_0000419	42.50804	7.245688	0.170455	0.009035	Down	sense-overlapping	RAB3IP
hsa_circ_0001402	33.81321	5.175492	0.153061	0.009594	Down	sense-overlapping	TBC1D1
hsa_circ_0008274	55.06723	8.280787	0.150376	0.004173	Down	sense-overlapping	UGGT2
hsa_circ_0002402	9.660918	0	0.00001	0.038795	Down	sense-overlapping	ECE1
hsa_circ_0012144	11.5931	0	0	0.017556	Down	sense-overlapping	ERI3

hsa_circ_0053535	12.55919	0	0	0.012138	Down	exonic	BIRC6
hsa_circ_0003910	9.660918	0	0	0.038795	Down	intergenic	
hsa_circ_0065299	14.49138	0	0	0.006034	Down	sense-overlapping	SMARCC1
hsa_circ_0005023	10.62701	0	0	0.025765	Down	sense-overlapping	STAG1
hsa_circ_0006248	9.660918	0	0	0.038795	Down	sense-overlapping	TRA2B
hsa_circ_0072081	10.62701	0	0	0.025765	Down	sense-overlapping	MTMR12
hsa_circ_0072386	9.660918	0	0	0.038795	Down	sense-overlapping	HMGCS1
hsa_circ_0001833	9.660918	0	0	0.038795	Down	sense-overlapping	MROH1
hsa_circ_0003537	9.660918	0	0	0.038795	Down	sense-overlapping	FOCAD
hsa_circ_0006276	39.60976	0	0	1.58E-05	Down	sense-overlapping	ANXA7
hsa_circ_0008148	10.62701	0	0	0.025765	Down	sense-overlapping	BTAF1
hsa_circ_0019170	11.5931	0	0	0.017556	Down	sense-overlapping	EXOC6
hsa_circ_0008189	9.660918	0	0	0.038795	Down	sense-overlapping	C11orf30
hsa_circ_0000555	9.660918	0	0	0.038795	Down	sense-overlapping	YLPM1
hsa_circ_0007979	9.660918	0	0	0.038795	Down	sense-overlapping	DYNC1H1
hsa_circ_0001172	10.62701	0	0	0.025765	Down	intergenic	
hsa_circ_0005386	10.62701	0	0	0.025765	Down	sense-overlapping	NF2

Figure S1: The correlation of potential target mRNAs of hsa_circ_0000567 with overall survival of lung cancer patients.

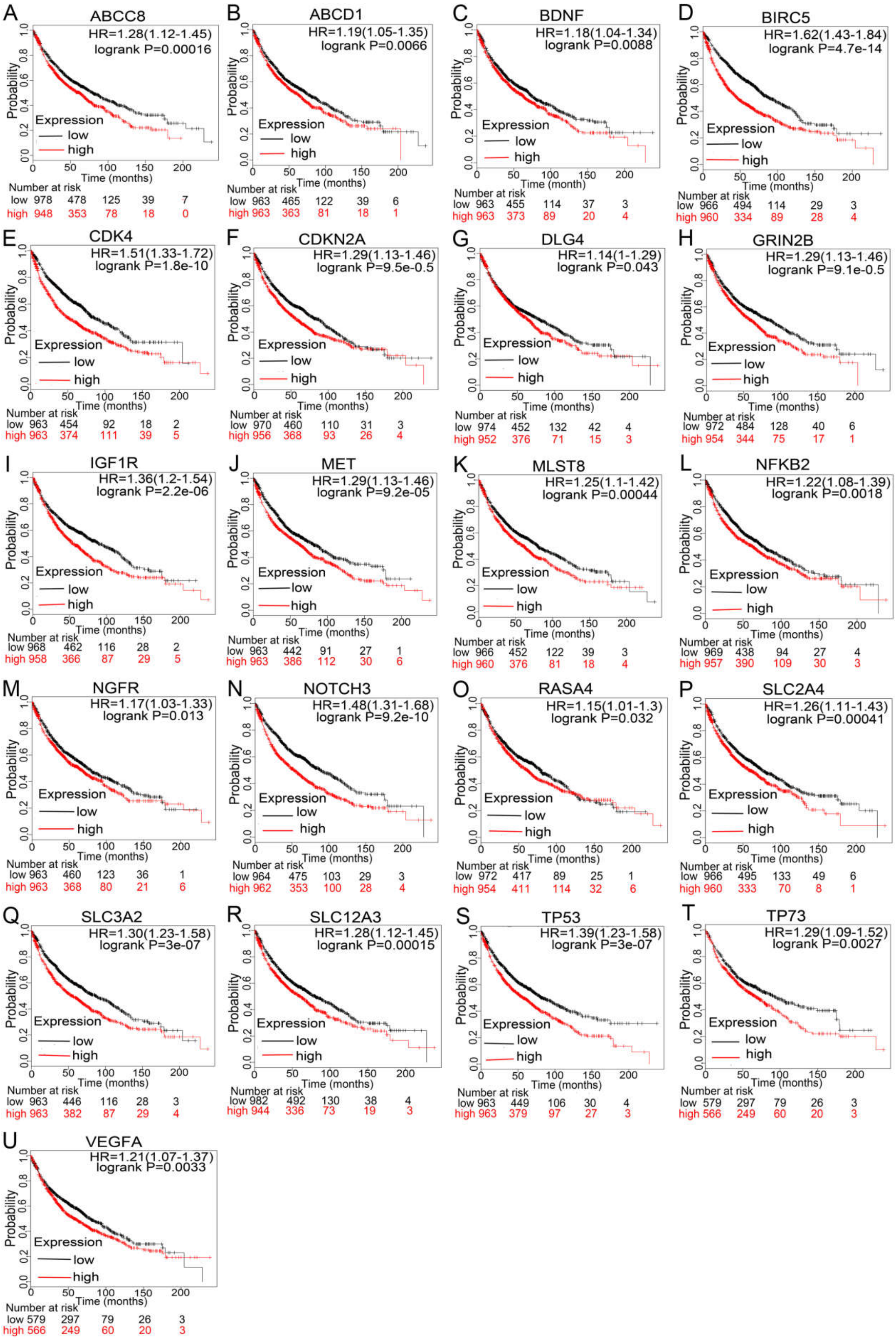

Figure S2: The correlation of potential target mRNAs of hsa_circ_0006867 with overall survival of lung cancer patients.

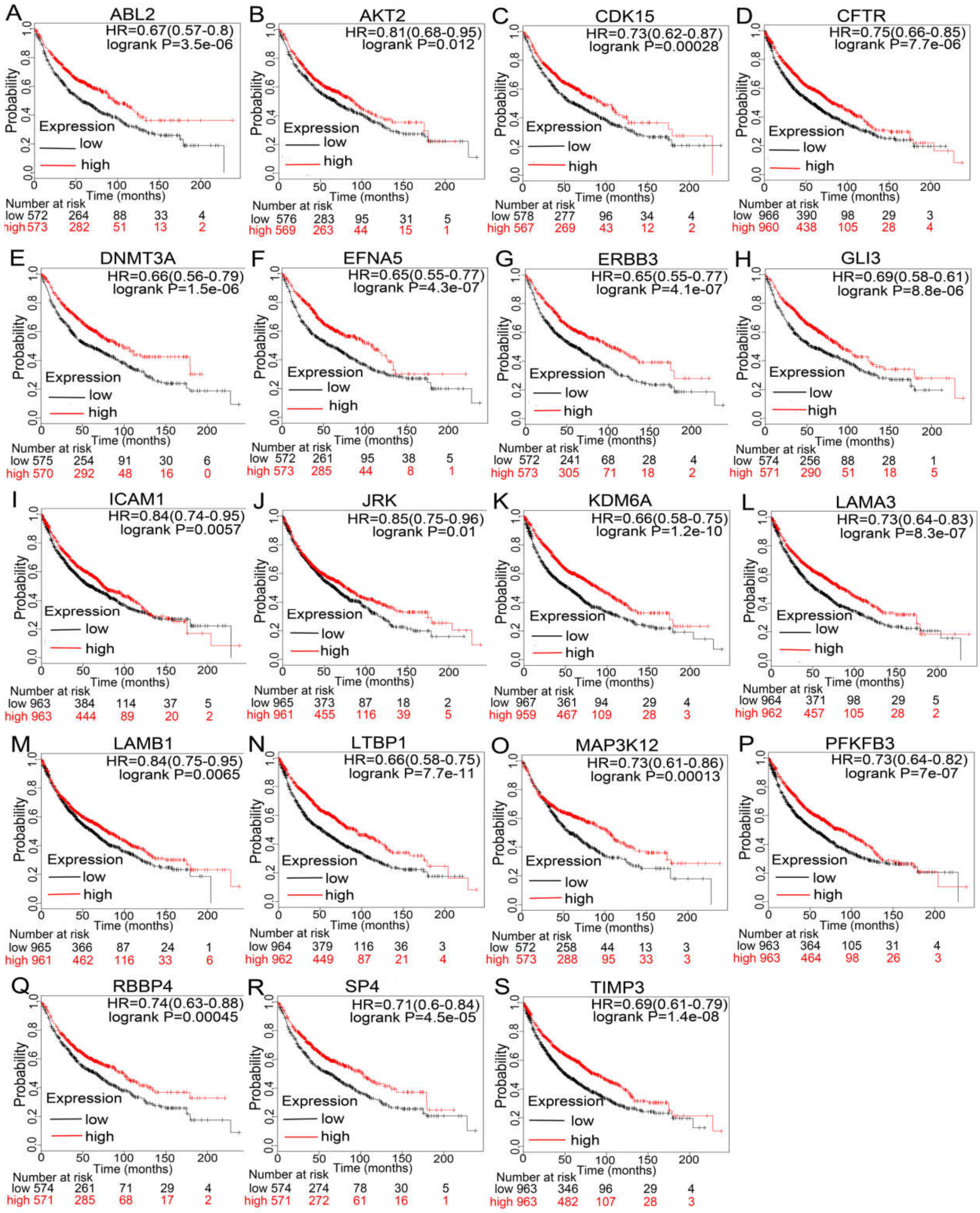

Supplement: Supplementary file 1 — Supplementary figures and tables. [file jcav11p3816s1.pdf]
